# Changes in dominant *Escherichia coli* and antimicrobial resistance after 24 hr in fecal matter

**DOI:** 10.1002/mbo3.643

**Published:** 2018-06-12

**Authors:** Sofía Barrera, Paul Cardenas, Jay P. Graham, Gabriel Trueba

**Affiliations:** ^1^ Microbiology Institute Universidad San Francisco de Quito Quito Ecuador; ^2^ Public Health Institute Oakland California

**Keywords:** antimicrobial resistance, Chicken, Clonal, *E. coli*, fecal matter, resistance genes

## Abstract

Intestinal bacteria carry antimicrobial resistance (AMR) genes in mobile genetic elements which have the potential to spread to bacteria in other animal hosts including humans. In fecal matter, *Escherichia coli* can continue to multiply for 48 hr after being excreted, and in certain environments, *E. coli* survive long periods of time. It is unclear the extent to which AMR in *E. coli* changes in the environment outside of its host. In this study, we analyzed changes in the population structure, plasmid content, and AMR patterns of 30 *E. coli* isolates isolated from 6 chickens (cloacal swabs), and 30 *E. coli* isolates from fecal samples (from the same 6 chickens) after 24 hr of incubation. Clonality of isolates was screened using the *fumC* gene sequence and confirmed in a subset of isolates (*n* = 14) by multi‐locus sequence typing. Major shifts in the population structure (i.e., sequence types) and antibiotic resistance patterns were observed among the numerically dominant *E. coli* isolates after 24 hr. Four *E. coli* clones isolated from the cloaca swabs and the corresponding fecal samples (after 24 hr incubation) showed different antibiotic resistance patterns. Our study reveals that fecal matter in the environment is an intermediate habitat where rapid and striking changes occur in *E. coli* populations and antibiotic resistance patterns.

## 1 INTRODUCTION

The misuse of antibiotics in domestic animals, as well as in human medicine, is an important contributor to the current crisis of antimicrobial resistance (AMR) (Gelband et al., 2015). In the United States, for example, it has been estimated that 80% of antibiotics are used in food‐animal production (Gelband et al., 2015). The human acquisition of AMR from domestic animals could occur by consuming fecally contaminated food‐animal products, contaminated water, having contact with uncooked meat or poultry, contact with domestic animals or contact with contaminated environments (Berg et al., 2017; Gelband et al., 2015; Johnson et al., 2008; Ercumen et al., 2017). The risk of AMR transmission from microbiota in domestic animals to human microbiota through contaminated environments is present in developing countries and industrialized countries mainly due to agricultural practices where animal waste is used as a fertilizer (Graham et al., 2009; Graham et al., 2017; Heuer, Schmitt, & Smalla, 2011; Pehrsson et al., 2016 Seiffert et al., 2013).

Transmission of AMR trough the environment is probably more important in *E. coli* than any other member of the microbiota because *E. coli*: (1) is the most abundant facultative aerobe in the intestines which can not only tolerate oxygen but also grow for a period of time in fecal matter excreted from the host (Russell and Jarvis, 2001; Vasco, Spindel, Carrera, Grigg, & Trueba, 2015), (2) it can remain viable in the environment (secondary habitat) for extended periods of time (Savageau, 1983), and (3) it is highly active in horizontal transfer of AMR genes (Berg et al., 2017; Dobiasova & Dolejska, 2016; Johnson et al., 2012; Liu et al., 2016).

Antimicrobial resistance in *E. coli* is regarded as major threat to public health (Gelband et al., 2015), yet little is known about how *E. coli*, especially drug‐resistant *E. coli*, changes once it leaves its host. Some studies have found that the composition of *E. coli* populations changes when fecal matter is exposed to the environment (Gordon, Bauer, & Johnson, 2009; Whittam, 1989). It is unknown, however, whether the numerically dominant (Lautenbach, Bilker, Tolomeo, & Maslow, 2008) and antimicrobial resistant *E. coli* strains in fresh fecal matter remain dominant over time, or whether AMR patterns in the population of *E. coli* present at the time of excretion impose a fitness cost once *E. coli* leaves the intestine. Given the limited research on this important public health topic, we investigated changes in the *E. coli* population, plasmids and AMR genes, when fecal matter remains in the environment outside the host for 24 hr. Understanding changes in AMR and mobile genetic elements (MGEs) of Enterobacteriaceae, such as *E. coli*, in fecal waste could help in developing treatment standards for this waste that may carry drug‐resistant bacteria.

## 2 EXPERIMENTAL PROCEDURES

### 2.1 Sample collection

Six chickens were obtained from two farms (1 broiler and 2 Turken chickens from one farm and 3 Turken chickens from another farm), and each chicken was labeled for the study: A, B, C, D, E, and F. The chickens were housed together and raised without antibiotics for 2‐months prior to the experiment. A cloacal swab from each chicken was obtained and inoculated directly onto MacConkey agar plates and incubated at 37°C for 24 hr. We also obtained a fecal sample from each chicken, placed it on a Petri dish, incubated it for 24 hr at room temperature and inoculated it onto a MacConkey agar plate as stated earlier. A small wet cotton ball was placed in each of the Petri dishes in a separate compartment to prevent dehydration of the fecal sample and to warrant more reproducible results. Five lactose fermentative colonies were obtained from each MacConkey agar plate, and each colony was tested for Beta‐glucuronidase activity using Chromocult Agar (Merck, Darmstadt, Germany). Isolates were then frozen at −80°C for further testing (Vasco et al., 2015). These five colonies represented the most abundant *E. coli* clones (i.e., dominant clones) in each cloacal or fecal matter sample (Lautenbach et al., 2008). *E. coli* isolates were labeled with a letter representing the chicken of origin (A to F) followed by an “i” for isolates from cloaca and “f” for isolates from fecal matter; the isolates from either cloaca or feces were numbered 1 to 5. All animal protocols were approved by the Animal Bioethics Committee of Universidad San Francisco de Quito.

### 2.2 Antimicrobial susceptibility test

All the isolated strains were tested for their susceptibility to antimicrobials using the Kirby Bauer technique (i.e., disc diffusion in Muller Hinton Agar) (Bauer, Kirby, Sherris, & Turck, 1966). The following antibiotics were tested: ampicillin (10 μg‐AM), amoxicillin + clavulanic acid (20 μg‐AMC), cefotaxime (30 μg‐CTX), cefalotin (30 μg‐CF), chloramphenicol (30 μg‐C), ciprofloxacin (5 μg‐CIP), trimethoprim sulfamethoxazole (5 μg‐SXT), gentamicin (10 μg‐GM), tetracycline (30 μg‐TE), and enrofloxacin (5 μg‐ENO). After 24 hr of incubation at 37°C, zones of inhibition were measured and resistance was determined according to the Clinical and Laboratory Standards Institute guidelines (CLSI, 2015).

### 2.3 Genotyping

DNA extraction was performed using a Promega Wizard Genomic DNA Purification Kit (Madison, WI, USA) following the procedure suggested by the manufacturer. Isolates from each chicken were initially screened using the *fumC* DNA sequence to identify potential clones (Vasco, Graham, & Trueba, 2016). Due to economic constraints, only identical strains, based on the fumC sequence, that displayed AMR pattern changes (which could indicate different bacterial clones), were subjected to complete multi‐locus sequence typing (MLST) to confirm clonality using the primers for the genes *adk*,* girB*,* icd*,* mdh*,* purA*, and *recA* (Wirth et al., 2006). PCR conditions were: 120 s at 95°C, 30 cycles of 60 s at 95°C, 60 s at 55°C (*adk*,* girB*,* icd*,* mdh*,* purA*, and *recA*) or 60°C (*fumC*) and 120 s at 72°C, and a final extension of 5 min at 72°C. Ampicons were sequenced using Sanger's method at Functional Biosciences (Madison, WI, USA). The phylogenetic group was analyzed using a previously published PCR protocol that used the primers pairs ChuA.1, ChuA.2; YjaA.1, YjaA.2; TspE4C2.1 TspE4C2 and the conditions 5 min at 94°C and 30 cycles of 30 s at 94°C, 30 s at 55°C, and 30 s at 72°C; and extension of 7 min at 72°C (Clermont, Bonacorsi, & Bingen, 2000).

### 2.4 Conjugation

We selected isolates belonging to the same genotype but having different AMR patterns after fecal incubation, to further analyze plasmids. Azide‐resistant *E. coli* strain J53 was used as the receptor bacteria for all conjugation assays (Yi, Cho, Yong, & Chun, 2012). Initially isolates were inoculated in nutrient agar, and 24 hr after incubation, inoculated into 5 ml of Tryptic Soy Broth and incubated 24 hr at 37°C (Kruse & Sørum, 1994). After incubation, receptor and donor strains were mixed together in one tube and incubated 24 hr at 37°C. Muller Hinton media with 2% sodium azide and tetracycline, cefazolin or trimethoprim sulfamethoxazole was used to select trans‐conjugants. AMR transfer was confirmed by Kirby Bauer antibiotic susceptibility testing. Transferred plasmids were characterized using a PCR‐based Replicon Typing kit (Diatheva, Viale Piceno, Italia). PCR reactions were prepared following the manufacturer's instructions (Carattoli et al., 2005). The PCR parameters were: denaturation 95°C for 10 min; 25 cycles of amplification (95°C for 60 s, 60°C for 30 s, and 72°C for 60 s) and a final incubation at 72°C for 5 min. A 2.5% agarose gel containing ethidium bromide was used for electrophoresis.

## 3 RESULTS

### 3.1 Numerically dominant *E. coli*

**Figure 1:**
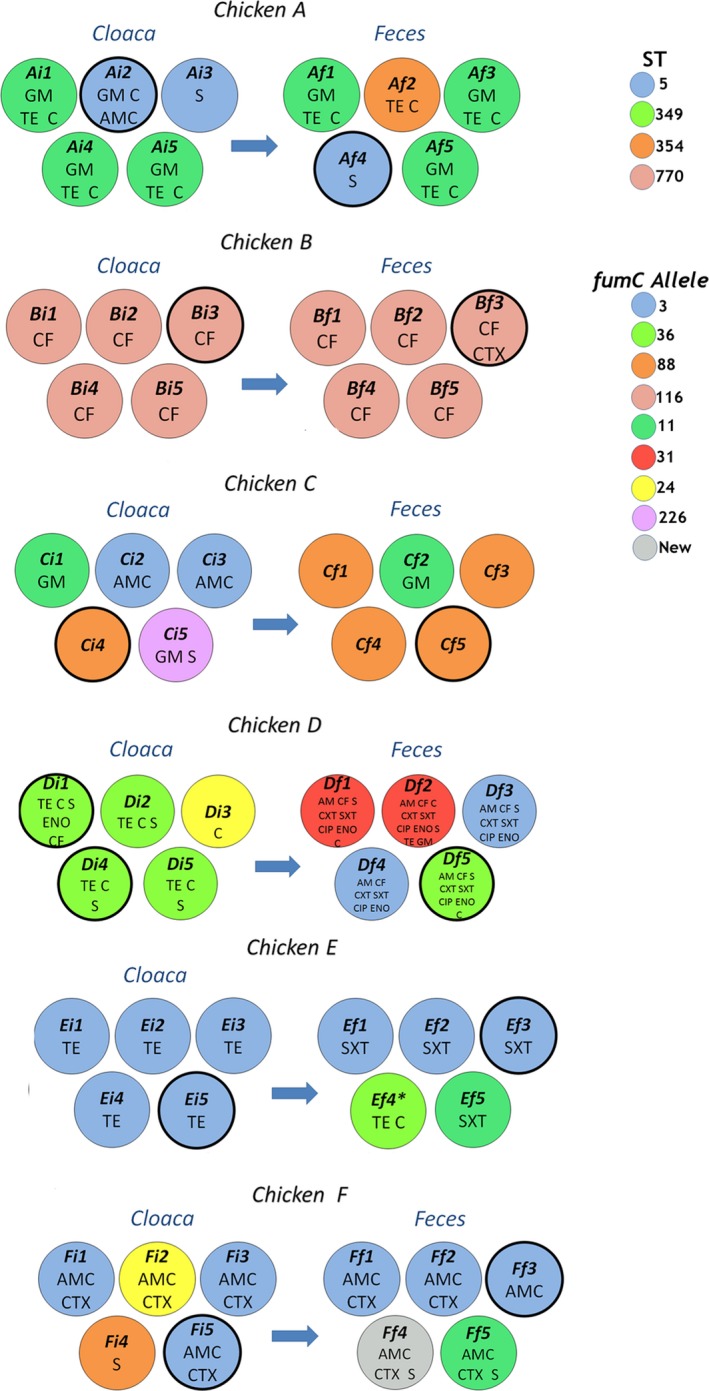
*Escherichia coli* isolates obtained from six chickens (A–F) from either cloaca (i) or after 24 hr incubation in fecal matter (f). Colors in the circles indicated sequence types (STs) or *fumC s*equences associated with a ST; (information at the right upper corner). Letters inside circles indicate antibiotic resistance that was not shared among isolates from the same animal. Thicker circumference lines represent strains that were subjected to a full multi‐locus sequence typing analysis and conjugation experiments

A total of 60 *E. coli* isolates were obtained from 6 chickens; 30 from cloacal swabs and 30 from corresponding fecal samples after 24‐hr of incubation. We found that 53 isolates belonged to phylogroup B2 and 7 isolates to phylogroup D (Di1, Di2, Di3, Di4, Di5, Df1, Df2; which corresponded to clones ST349, *fumC*24 and *fumC*31) and 1 isolate from chicken E (Ef4, *fumC*36) which belonged to phylogroup D. All the same STs (and *fumC* alleles) were part of the same phylogenetic groups. Sequence analyses of *fumC* gene (and confirmation using all seven MLST genes for 14 of the isolates) showed that isolates from two samples (chickens C and D) had striking differences in the *E. coli* clonal composition. In cloacal sample C, 1 out of 5 colonies was ST354 but after 24 hr incubation 4 out of 5 colonies were ST354; in the cloacal sample D, 4 out of 5 colonies were ST349, but after 24 hr incubation only 1 out of 5 colonies were ST349 (Figure 1, Table 1). In addition, clones not found in cloacal sample D were the most abundant after 24 hr incubation in fecal matter (Df1–Df4): Fecal samples A, E, and F showed modest change but some clones not found in cloacal samples, emerged after 24 hr of fecal matter incubation. Fecal and cloacal samples from chicken B showed no difference in clonal composition (Figure 1). These data suggest a different aptitude of some *E. coli* strains to replicate in fecal matter. In three chickens (A, E, F) *E. coli* ST5 was present, which may indicate that this clone could behave as numerically dominant in different chickens; chickens may have become colonized with this clone from their farms or ST5 may have been transferred among the chickens during the 2 months that the chickens were housed together (Table 1).

**Table 1:** Multi‐locus sequence typing, antibiotic resistance profile and plasmid analysis of selected *Escherichia coli* isolates obtained from six chickens (A–F) from either cloaca (i) or fecal matter (f)

Strain	ST	Phylogroup	Antibiotic resistance	Transconjugant resistance	Replicon typing
Af4	5	B2	AM CF CIP CTX ENO SXT	ND	ND
Ai2	5	B2	AM AMC C CF CIP CTX ENO GM SXT TE	ND	ND
Bf3	770	B2	C CIP CTX ENO TE	C CIP CTX ENO TE	I2 F/B L K FII X1
Bi3	770	B2	C CF CIP ENO TE	C CIP ENO TE	I2 B/O F/B P FII5 A/C FII5 K FII
Ci4	354	B2	AM C CF CIP CTX ENO SXT TE	ND	ND
Cf5	354	B2	AM C CF CIP CTX ENO SXT TE	ND	ND
Di4	349	D	C TE	C TE	I2 F/B X1 Y K FII
Di1	349	D	C CF ENO TE	C CF TE	I2 B/O F/B I1γ K A/C FII
Df5	349	D	AM C CF CIP CTX ENO SXT	AM C CF CIP CTX ENO SXT	I2 B/O F/B A/C I1γ P FII5 X1 K FII
Ei5	5	B2	AM CF CIP CTX ENO TE	ND	ND
Ef3	5	B2	AM CF CIP CTX ENO SXT	ND	ND
Fi5	5	B2	AM AMC C CF CIP CTX ENO GM SXT TE	AM AMC C CF CIP CTX ENO SXT TE	I1α I2 FIB FII
Ff3	5	B2	AM AMC C CF CIP ENO GM SXT TE	AM AMC C CF CIP ENO SXT TE	I1α I2 FIB FII B/O A/C

ND, not determined.

### 3.2 Phenotype resistance

Two *E. coli* potential clones (ST349, and ST5) isolated from cloacal swabs showed different patterns of antimicrobial resistance when isolated from fecal samples after 24 hr incubation. An isolate belonging to ST349, Df5, showed additional resistance to AM, CF,CIP, CTX, ENO, STX, and 3 isolates belonging to ST5 (Ef1, Ef2, and Ef3) showed additional resistance to STX (Figure 1). Conversely, other *E. coli* clones (ST349 and ST5) showed less types of antimicrobial resistances after incubation for 24 hr in fecal matter; ST349 (Df5) and three colonies belonging to ST5 (Ef1‐Ef3) from fecal matter, did not have TET resistance and isolate F3 did not have CTX resistance (Figure 1).

### 3.3 Plasmid content

In samples from chickens D and F, *E. coli* isolates belonging to the same ST (ST349 and ST5) showed different replicons depending whether the isolates came from the cloaca or fecal matter incubated for 24 hr. Cloacal isolates belonging to ST349 (Di1 and Di4) shared four replicons but Di1 had replicons B/O, I1γ, A/C which were absent in Di4, whereas Di4 had replicons X1, Y which were absent in Di1; additionally an isolate from fecal matter after 24 hr (ST349, Df5) had all replicons found in Di1 plus replicons P, FII5, X1 (absent in Di1) (Figure 1, Table 1). Similarly, a cloacal isolate belonging to ST5 (Fi5) had replicons I1α, I2, FIB, FII, whereas the isolate from fecal matter after 24 hr (ST5, Ef3) had additional replicons B/O and A/C (Figure 1, Table 1).

## 4 DISCUSSION

### 4.1 Changes in numerically dominant *E. coli*

The results of this analysis suggest that *E. coli* present in fresh feces can rapidly go through important changes, in terms of AMR, population structure and plasmid content, after the fecal matter enters the aerobic environment. Previous reports have indicated that the environment outside the host could select stress‐tolerant strains of *E. coli* (Bergholz, Noar, & Buckley, 2011; Gordon et al., 2009). Our study suggests that some of these strains that are present in animal intestines and can start emerging 24 hr after fecal deposition. These results are consistent with previous reports revealing that the population structure of *E. coli* in fresh feces differs from *E. coli* populations isolated from the non‐host environment (Anderson, Whitlock, & Harwood, 2005; Bergholz et al., 2011; Gordon et al., 2009; Whittam, 1989). Unlike previous reports (Bergholz et al., 2011; Ihssen et al., 2017), we posit that *E. coli* population from the intestine is composed of various lineages possessing different abilities to grow in fecal matter once excreted; some lineages may grow better than others in the presence of oxygen. There is a possibility, however, that some of our observations may reflect high mortality rates of certain *E. coli* clones. We found that all isolates belonged to phylogroups B2 and D, which have been found previously in domestic animals and the environment (Stoppe et al., 2017); however, the B2 phylogroup is not common in environments outside of the host, and neither B2 nor D phylogroups have been found to be common in poultry (Blyton et al., 2015).

Even though the chickens were housed together for 2 months, the majority (*n* = 18, 60%) of *E. coli* isolates from cloacal swabs seemed to be distinct in the different chickens (Figure 1, Table 1), this finding may indicate that only a few clones (e.g., ST5) have the ability to become dominant in different animals of the same species; it is possible that chickens may have arrived already colonized by *E. coli* with the same ST and these isolates may not be clones.

### 4.2 Phenotypic resistance and plasmids

Antimicrobial resistance profiles of the dominant *E. coli* strains also changed over 24 hr in the fecal matter; Two isolates with same ST (potential clones from 2 different chickens) showed resistance to more antimicrobials, whereas 3 isolates with same ST (potential clones from 3 different chickens) showed resistance to fewer antimicrobials (Figure 1). We argue that fitness cost or fitness advantage of certain plasmids may be different in the intestines compared to an aerobic environment (i.e., isolates with the same ST with different plasmids may be present in chicken intestines). Alternatively, horizontal gene transfer of plasmids in the feces could have been the mechanism for the acquisition of antimicrobial resistance (Figure 1, isolate Df5), as conjugation in fecal matter has been described previously (Kruse & Sørum, 1994). Plasmid loss may have occurred in the feces exposed to the environment for 24 hr, given that it is potentially a less hospitable environment (Stanisich & Bennett, 1984); however, these explanations may require that rates of conjugation (and plasmid loss) to be higher than under lab conditions (Wan, Varshavsky, Teegala, McLawrence, & Goddard, 2011). This study also suggests an incredibly dynamic plasmid transfer, which most likely occurs in the intestines; the number of *E. coli* cells that are transferred from the donor animal to the animal becoming colonized may be very small (Besser, Richards, Rice, & Hancock, 2001) which makes it unlikely that a chicken acquired two members of the same clone with different plasmids. All plasmid replicons found in this study have been described in other studies containing similar antimicrobial resistance in *E. coli* from chickens (Dobiasova & Dolejska, 2016; Johnson et al., 2012; Liu et al., 2016).

Our study has some limitations. First, we collected fresh fecal samples by swabbing the cloaca instead of obtaining a fecal sample. It could be argued that the swab may have collected a different population of *E. coli* than that found in feces. However, given that we identified the same clones in both sample types, may indicate that these two populations are similar. Other authors have also indicated that both populations are equivalent (Lautenbach et al., 2005). Second, we analyzed a small number of *E. coli* isolates from each of the twelve fecal samples. We, however, were interested in the changes that occur in the numerically dominant *E. coli* clones and selecting five colonies has been shown previously to represent the most abundant clones in the intestine (Lautenbach et al., 2008). Finally, in our experiments, fecal matter was maintained moist, therefore our results may be applicable only to fecal matter in humid environments.

## 5 CONCLUSIONS

Recent studies have provided additional and strong evidence that antimicrobial resistant *E. coli* and plasmids containing AMR genes are being transferred from retail chicken meat to people (Berg et al., 2017). Transmission of antibiotic resistant *E. coli* (and MGEs coding for antimicrobial resistance) may also occur through fecally contaminated environments, especially in developing countries (Graham et al., 2017; Pehrsson et al., 2016). Our study provides evidence that changes in *E. coli* bacterial population and antibiotic resistance profiles occur very fast after *E. coli* leaves the animal host, a phenomenon that may have important consequences for the dissemination of some AMR bacteria and genes through the environment. Our study highlights a need for more research on this topic, including the factors that cause the change in the frequency of some *E. coli* clones. It will be important to know if some factors, such as plasmid composition or bacterial diversity in fecal matter outside the host, are playing a role in this phenomenon.

## CONFLICT OF INTEREST

The authors declare no conflict of interest.
