# Supplementary material for: Changes in dominant Escherichia coli and antimicrobial resistance after 24 hr in fecal matter
Source: Microbiologyopen. 2018 Jun 12;8(2):e00643. doi: 10.1002/mbo3.643 (PMC6391265; doi:10.1002/mbo3.643)
Supplement: Supplementary file 1 [file MBO3-8-e00643-s001.docx]

Table S1: Characteristics of all isolates from chickens cloacal samples (i) and fecal samples (f).

| **Strain** | **Phylogroup** | **ST** | **fum C** | **Antibiotic resistance** |
| --- | --- | --- | --- | --- |
| Ai1 | B2 | ND | 11 | AM C CF CIP CTX ENR GM SXT TE |
| Ai2 | B2 | 5 | 3 | AM AMC C CF CIP CTX ENR GM SXT |
| Ai3 | B2 | ND | 3 | AM CF CIP CTX ENR S SXT |
| Ai4 | B2 | ND | 11 | AM C CF CIP CTX ENR GM SXT TE |
| Ai5 | B2 | ND | 11 | AM C CF CIP CTX ENR GM SXT TE |
| Af1 | B2 | ND | 11 | AM C CF CIP CTX ENR GM SXT TE |
| Af2 | B2 | ND | 88 | AM C CF CIP CTX ENR SXT TE |
| Af3 | B2 | ND | 11 | AM C CF CIP CTX ENR GM SXT TE |
| Af4 | B2 | 5 | 3 | AM CF CIP CTX ENR S SXT |
| Af5 | B2 | ND | 11 | AM C CF CIP CTX ENR GM SXT TE |
| Bi1 | B2 | ND | 116 | C CIP CF ENR TE |
| Bi2 | B2 | ND | 116 | C CIP CF ENR TE |
| Bi3 | B2 | 770 | 116 | C CIP CF ENR TE |
| Bi4 | B2 | ND | 116 | C CIP CF ENR TE |
| Bi5 | B2 | ND | 116 | C CIP CF ENR TE |
| Bf1 | B2 | ND | 116 | C CIP ENR TE |
| Bf2 | B2 | ND | 116 | C CIP ENR TE |
| Bf3 | B2 | 770 | 116 | C CIP CTX ENR TE |
| Bf4 | B2 | ND | 116 | C CIP ENR TE |
| Bf5 | B2 | ND | 116 | C CIP ENR TE |
| Ci1 | B2 | ND | 11 | AM C CF CIP CTX ENR GM SXT TE |
| Ci2 | B2 | ND | 3 | AM AMC C CF CIP CTX ENR GM SXT TE |
| Ci3 | B2 | ND | 3 | AM AMC C CF CIP CTX ENR GM SXT TE |
| Ci4 | B2 | 354 | 88 | AM C CF CIP CTX ENR SXT TE |
| Ci5 | B2 | ND | 266 | AM C CF CIP CTX ENR GM S SXT TE |
| Cf1 | B2 | ND | 88 | AM C CF CIP CTX ENR SXT TE |
| Cf2 | B2 | ND | 11 | AM C CF CIP CTX ENR GM SXT TE |
| Cf3 | B2 | ND | 88 | AM C CF CIP CTX ENR SXT TE |
| Cf4 | B2 | ND | 88 | AM C CF CIP CTX ENR SXT TE |
| Cf5 | B2 | 354 | 88 | AM C CF CIP CTX ENR SXT TE |
| Di1 | D | 349 | 36 | C CF ENR TE S |
| Di2 | D | ND | 36 | C S TE |
| Di3 | D | ND | 24 | C |
| Di4 | D | 349 | 36 | C S TE |
| Di5 | D | ND | 36 | C S TE |
| Df1 | D | ND | 31 | AM C CF CIP CTX ENR TE S |
| Df2 | D | ND | 31 | AM C CF CIP CTX ENR S SXT TE |
| Df3 | B2 | ND | 3 | AM CF CIP CTX ENR S SXT |
| Df4 | B2 | ND | 3 | AM CF CIP CTX ENR S SXT |
| Df5 | D | 349 | 36 | AM C CF CIP ENR S SXT TE |
| Ei1 | B2 | ND | 3 | AM CF CIP CTX ENR S SXT |
| Ei2 | B2 | ND | 3 | AM CF CIP CTX ENR S TE |
| Ei3 | B2 | ND | 3 | AM CF CIP CTX ENR S TE |
| Ei4 | B2 | ND | 3 | AM CF CIP CTX ENR S TE |
| Ei5 | B2 | 5 | 3 | AM CF CIP CTX ENR S TE |
| Ef1 | B2 | ND | 3 | AM CF CIP CTX ENR S SXT |
| Ef2 | B2 | ND | 3 | AM CF CIP CTX ENR S SXT |
| Ef3 | B2 | 5 | 3 | AM CF CIP CTX ENR S SXT |
| Ef4 | D | ND | 36 | C TE |
| Ef5 | B2 | ND | 11 | AM CF CIP CTX ENR S SXT |
| Fi1 | B2 | ND | 3 | AM AMC C CF CIP CTX ENR GM SXT TE |
| Fi2 | B2 | ND | 24 | AM AMC C CF CIP CTX ENR GM SXT TE |
| Fi3 | B2 | ND | 3 | AM AMC C CF CIP CTX ENR GM SXT TE |
| Fi4 | B2 | ND | 88 | AM C CF CIP ENR GM SXT TE |
| Fi5 | B2 | 5 | 3 | AM AMC C CF CIP CTX ENR GM SXT TE |
| Ff1 | B2 | ND | 3 | AM AMC C CF CIP CTX ENR GM SXT TE |
| Ff2 | B2 | ND | 3 | AM AMC C CF CIP CTX ENR GM SXT TE |
| Ff3 | B2 | 5 | 3 | AM AMC C CF CIP ENR GM SXT TE |
| Ff4 | B2 | ND | NEW | AM AMC C CF CIP CTX ENR GM S SXT TE |
| Ff5 | B2 | ND | 11 | AM AMC C CF CIP CTX ENR GM S SXT TE |

ND= Not determined
